# *Iranotrichia* gen. n., a new genus of Scenopinidae (Diptera) from Iran, with a key to window fly genera of the world

**DOI:** 10.3897/zookeys.138.1821

**Published:** 2011-10-19

**Authors:** Shaun L. Winterton, Babak Gharali

**Affiliations:** 1 California State Collection of Arthropods, Plant Pest Diagnostics Center, California Department of Food & Agriculture, Sacramento, California, USA; 2 Department of Entomology, Ghazvin Research Center for Agriculture and Natural Resources, Ghazvin, IRAN.

**Keywords:** Therevoid clade, Asiloidea, Scenopinidae, key

## Abstract

An unusual new genus of Scenopinidae, *Iranotrichia* gen. n., comprising two newly discovered species (*Iranotrichia insolita* **sp. n.** and *Iranotrichia nigra* **sp. n.**), is described from Iran. *Iranotrichia* **gen. n.** are scenopinine window flies with a habitus reminiscent of certain bee fly genera (Bombyliidae), based on colouration and elongate mouthparts and antennae. The phylogenetic placement of this distinctive new genus is discussed and a dichotomous key to world genera is presented. The genus name *Kelseyana* **nom. n.** is proposed to replace *Caenoneura* Kröber, 1924, which was found to be preoccupied by Thomson (1870: 270) (Hymenoptera) and Kirby (1890: 136) (Odonata).

## Introduction

Window flies (Diptera: Scenopinidae) are a small family (ca. 420 species in 26 genera) of flies with an adult body size rarely exceeding 5.0 mm. Scenopinids are distributed throughout all major biogeographical regions, and with few exceptions, most genera are confined to a single biogeographical region (Kelsey, 1973). This family is placed in the therevoid clade, comprising families such as Therevidae (stiletto flies), Apsilocephalidae and Evocoidae (Winterton, 2008; Trautwein et al. 2010). A close relationship between Scenopinidae and Therevidae has been previously identified based on the secondarily segmented characteristic of the larval abdomen (Woodley 1989).

*Iranotrichia* gen. n. is described herein from northern Iran, comprising two species, *Iranotrichia insolita* sp. n. and *Iranotrichia nigra* sp. n. This new genus is morphologically very similar to other scenopinine genera such as *Metatrichia* Coquillett, 1900, *Kelseyana* nom. n. (= *Caenoneura* Kröber, 1923) and *Pseudomphrale* Kröber, 1913. This group of genera is differentiated by other scenopinine genera based on the following characteristics: wing vein M_1 _fused to vein R_5_ before wing margin, mouthparts well developed, scutum with extensive pile (often scale-like), abdomen broad and flattened in both sexes with limited sexual dimorphism, male genitalia with gonocoxal apodemes relatively broad, and female acanthophorite spines absent. *Metatrichia* is a cosmopolitan genus containing 16 extant and one fossil species. (Kelsey 1969; Krivosheina and Krivosheina 1999; Winterton and Woodley 2009; Yeates and Grimaldi 1995). The morphological differences between *Metatrichia* and *Pseudomphrale* are not clear, and the validity of this distinction has been questioned previously (Krivosheina and Krivosheina 1996; Winterton and Woodley 2009). *Iranotrichia* gen. n. is easily differentiated from all other scenopinine genera based on the characters above, as well as the elongate antennae and mouthparts, subterminal antennal style and greatly elongate distiphallus in the male genitalia. This new genus is described and figured herein, with a key to the 25 extant scenopinid genera of the world presented. The genus name *Kelseyana* nom. n. proposed to replace *Caenoneura* Kröber, which was found to be is preoccupied by Thomson (1870: 270) (Hymenoptera) and Kirby (1890: 136) (Odonata).

## Materials and Methods

Genitalia were macerated in 10% KOH at room temperature for one day to remove soft tissue, then rinsed in distilled water and dilute acetic acid, and dissected in 80% ethanol. Preparations were then placed into glycerine, with images made with the aid of a digital camera mounted on a stereomicroscope. Genitalia preparations were placed in glycerine in a genitalia vial mounted on the pin beneath the specimen. Terminology follows Winterton (2005) and Winterton and Woodley (2009). In contrast to the scenopinid subfamilies Proratinae and Caenotinae, the male terminalia of Scenopininae are rotated 180°. To avoid confusion with terminology and comparative homology, structures are described and labeled as they are in related flies with terminalia not rotated; therefore the ventral apodeme of the aedeagus described herein is physically located dorsally. The following collection acronyms are cited in the text: California Academy of Sciences, San Francisco, California, USA (CAS),California State Collection of Arthropods, Sacramento, California, USA (CSCA), Iranian Research Institute of Plant Protection, Tehran, Iran (IRIPP), and the National Museum of Natural History, Smithsonian Institution, Washington DC, USA (NMNH). Specimen images were taken at different focal points using a digital camera and subsequently combined into a serial montage image using Helicon Focus software. High-resolution digital images were deposited into Morphbank with embedded URL links within the document between descriptions and Morphbank images. All new nomenclatural acts and literature are registered in Zoobank (Pyle and Michel 2008).

## Taxonomy

Key to therevoid clade families and Scenopinidae genera of the world:

Note: *Metatrichia* and *Pseudomphrale* cannot be separated at this time and are included together in couplet 21.

**Table:** 

1	Wing with vein CuA_1_ arising from apex of cell *bm*; connected to discal cell by cross-vein m-cu; three medial veins present; male epandrium not medially divided	2
–	Wing with vein CuA_1_ integrated into at least part of posterior margin of discal cell; one or two (rarely three) medial veins present; male epandrium medially divided	3
2	Antennal style elongate and filamentous	Apsilocephalidae
–	Antennal style very short, often barely evident	Therevidae
3	Antennal flagellum comprised of bulbous base fused with a terminal filamentous style; vein CuA_2_ separate from A_1_ to wing margin	Evocoidae
–	Antennal flagellum shape variable, but never with an elongate terminal filament; vein CuA_2_ joining to A_1_, petiolate to wing margin	Scenopinidae: 4
4	Wing with two veins originating posteriorly from discal cell (M_1_ and CuA_1_); male genitalia rotated 180°	Scenopininae: 11
–	Wing with three (or rarely four) veins originating posteriorly from discal cell (M_1_, M_2_, M_3_ and CuA_1_); male genitalia not rotated	5
5	Costal vein extending around wing; sensory area on tergite 2 made up of two hemispherical regions of short setae; male genitalia with aedeagus and gonocoxal apodemes short (Nearctic)	*Caenotinae*: Caenotus Cole, 1923
–	Costal vein ending at vein R_5_; male genitalia with aedeagus and gonocoxal apodemes greatly elongate	Proratinae: 6
6	Abdominal tergite 2 setal patch absent; antennal flagellum abruptly turbinate with a tuft of apical setae; thickening of costal margin ending at or just beyond R_4_; abdomen largely white with brown terminalia (Nearctic)	*Caenotoides* Hall, 1972
–	Abdominal tergite 2 setal patch present; antennal flagellum cylindrical or tapered, without tuft of apical setae; thickening of costal margin ending at or just beyond R_5_; abdomen typically uniform black, brown or pale yellow	7
7	Mouthparts elongate; antennal flagellum cylindrical; elongate setae along posterior margin of female abdominal tergite 8; male wing with M_1 _much shorter than M_2_; male gonocoxites with medial spine projecting posteriorly; gonocoxal apodemes and aedeagus barely projecting anteriorly from gonocoxites (Neotropical: Argentina)	*Jackhallia* Nagatomi & Liu, 1994
–	Mouthparts length variable; antennal flagellum usually tapered, although sometimes cylindrical; setae along posterior margin of female abdominal tergite 8 short; male wing with M_1 _longer than or equal length of M_2_; male gonocoxites without medial spine; gonocoxal apodemes and aedeagus project anteriorly well beyond gonocoxites, sometimes greatly elongated	8
8	Abdominal tergite 2 setal patch rounded with very slight medial separation into two hemispheres; female tergite 8 with erect, elongate setae arranged in ring-like pattern; male aedeagus folded dorsally onto itself so that ejaculatory apodeme is projecting posteriorly (Afrotropical: Namibia)	*Cyrtosathe* Winterton & Metz, 2005
–	Abdominal tergite 2 setal patch as a single rounded or elongate patch; female tergite 8 without erect, elongate setae; male aedeagus extending anteriorly and not folded on itself	9
9	Antennal flagellum gradually narrowed apically, with thick apical style that is wider than apex of preceding segment; anterior margin of female eye not emarginate (Nearctic)	*Acaenotus* Nagatomi & Yanagida, 1994
–	Antennal flagellum only slightly tapered, with narrow apical, or more commonly subapical, style that is not wider than apex; anterior margin of female eye often triangularly emarginate just dorsal to base of antennae	10
10	Antennal flagellum with single segment (excluding apical style) (Palaearctic)	*Alloxytropus* Bezzi, 1925
–	Antennal flagellum two segmented (excluding apical style), apical segment is minute and similar shaped to style in some species (e.g. *Prorates frommeri* Hall, 1972) (Nearctic)	*Prorates* Melander, 1906
11	Wing vein M_1_ separate from vein R_5_ to wing margin (cell r_5_ open)	12
–	Wing vein M_1_ fused to vein R_5_ before wing margin (petiolate closed cell r_5_)	16
12	Wing vein M_1_ incomplete or terminating before wing margin	13
–	Wing vein M_1 _complete to wing margin	14
13	Male epandrium as two relatively short lobes; female with reduced spines on acanthophorite; female sternite 8 longer than tergite 8 (Australasian)	*Riekiella* Paramonov, 1955 (part)
–	Male epandrium as four elongate lobes; female acanthophorite spines elongate, slender; female tergite 8 and sternite 8 subequal (Australasian)	*Paramonova* Kelsey, 1970 (part)
14	Vein CuA_1_ terminating just beyond cell d; female sternite 8 with comb-like band of elongate setae (Afrotropical, Oriental)	*Seguyia* Kelsey, 1980
–	Vein CuA_1_ reaching or terminating just prior to posterior wing margin; female sternite 8 without distinct comb-like band of elongate setae	15
15	Head length generally longer than height (sometimes subequal); body elongate; abdomen elongate and cylindrical; reared from wood-boring beetle galleries (Afrotropical, Palaearctic, Oriental)	*Prepseudatrichia* Kelsey, 1969
–	Head length generally shorter than height; body relatively short; abdomen wide; reared from various habitats but not known from wood-boring beetle galleries (cosmopolitan)	*Scenopinus* Latreille , 1802
16	Mouthparts atrophied (Nearctic)	*Belosta*Hardy, 1944
–	Mouthparts well developed	17
17	Head generally longer than high; body glossy black with verrucous surface microsculpturing, without extensive setal pile; abdomen greatly elongate and cylindrical along entire length; reared from wood-boring beetle galleries or vertebrate nests	18
–	Head generally shorter than high; body glossy black or frequently with extensive pubescence, surface microsculpturing absent, often with extensive setal pile; abdomen short and flat, sometimes greatly elongate and tapered in female, never cylindrical along entire length; not known from wood-boring beetle galleries	19
18	Male epandrium approximately as long as high (lateral view); posterior margin of sternite 6 unmodified, without processes; male gonostyli with comb-like band of elongate setae; female cerci without tuft of strong spines; apex of cell r_5_ blunt (Nearctic, Neotropical)	*Pseudatrichia* Osten Sacken, 1877
–	Male epandrium distinctly shorter than high, band like (lateral view); truncated process along posterior margin of sternite 6; male gonostyli without elongate setae; female cerci with tuft of strong, ventrally directed spines; apex of cell r_5_acute (Australasian)	*Neopseudatrichia* Kelsey, 1969
19	Relatively robust bodied flies with broad, flat abdomen in both sexes (frequently large sized); sexes approximately equal sized	20
–	Relatively delicate flies with narrow tapered abdomen (usually with relatively small body size), particularly in female; abdomen much longer in female, displaying distinct sexual size dimorphism	22
20	M_1_ and composite R_5_+M_1_ vein abruptly bent anteriorly to join wing margin subapically along costa (Palaearctic)	*Kelseyana* nom. n.(= Caenoneura Kröber, 1923)
–	M_1_ and composite R_5_+M_1_ vein not bent anteriorly (Fig. 1A), joining margin at wing apex	21
21	Antennae greatly elongate and cylindrical; flagellum broadly rounded to truncate apically but not notched, style subterminal (Figs 1B-C); mouthparts greatly elongate; male distiphallus greatly elongate and coiled (Figs 4A-D); body reminiscent of bee fly (Bombyliidae) (Palaearctic: Iran)	*Iranotrichia* gen. n.
–	Antennae not elongate; flagellum ovate to quadrangular, notched apically with style terminal in notch; mouthparts rarely elongate; male distiphallus short and straight, rarely protruding beyond genitalic capsule; body not resembling bee fly	*Metatrichia* Coquillett, 1900 (Cosmopolitan) and *Pseudomphrale* Kröber, 1913 (Palaearctic)
22	Glossy black flies without pubescence; antennal flagellum pointed, not notched; female cerci with tuft of strong ventrally projecting setae (Palaearctic)	*Stenomphrale* Kröber, 1937
–	Body variously coloured with dense pubescence (rarely glossy black); antennal flagellum broad, notched apically; female cerci without tuft of strong setae	23
23	Wing with vein R_4_ branching from R_5_ along the basal half of cell r_5_; female acanthophorite spines well developed	24
–	Wing with vein R_4_ branching from R_5_ at halfway or along distal half of cell r_5_; female acanthophorite spines present, or reduced in size or shape, sometimes absent	25
24	Male distiphallus short and straight; male subepandrial sclerite not modified; female sternite 8 straight or slightly emarginate apically (Neotropical)	*Heteromphrale* Kröber, 1937
–	Male distiphallus relatively long and thread-like, highly reflexed basally so that basiphallus and ejaculatory apodeme are projecting dorsally or posteriorly; subepandrial sclerite with anterior projecting, blade-like extensions serving as aedeagal guides; female sternite 8 with rounded posterolateral lobes (Nearctic, Neotropical)	*Brevitrichia* Hardy, 1944
25	Female acanthophorite spines well developed (Afrotropical)	*Propebrevitrichia* Kelsey, 1969
–	Female acanthophorite spines absent, or greatly reduced in length or thickness (Australasian, Neotropical)	26
26	Female sternite 8 apically trilobate; male epandrium unmodified, without posterior or medial processes (Neotropical)	*Irwiniana* Kelsey, 1971
–	Female sternite 8 apically rounded or bilobate; male epandrium typically with multiple lobes and posterior or medial processes (Australasian)	27
27	Male epandrium with flange-like lobes internally	*Paratrichia* Kelsey, 1969
–	Male epandrium without flange-like lobes internally	28
28	Male epandrium as two relatively short lobes; female sternite 8 longer than tergite 8, apically pointed	*Riekiella* Paramonov, 1955 (part)
–	Male epandrium as four elongate lobes; female sternite 8 length subequal to tergite 8	*Paramonova* Kelsey, 1970 (part)

### Iranotrichia gen. n.

urn:lsid:zoobank.org:pub:066C7733-FC95-4532-9465-8B123D0BEB33

http://species-id.net/wiki/Iranotrichia

#### Type species:

*Iranotrichia insolita* sp. n.

#### Diagnosis.

**Figure 1.**
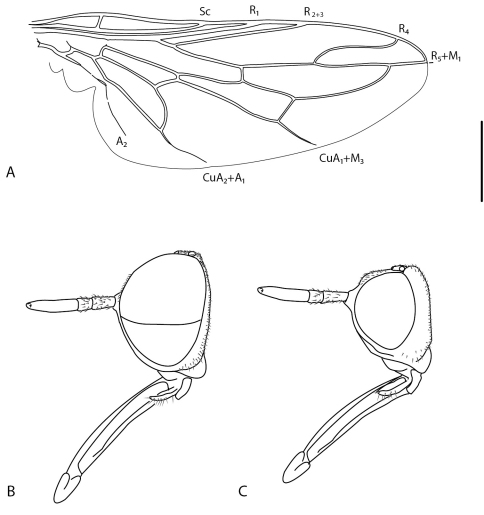
*Iranotrichia insolita* sp. n.: **A.** wing; **B.** male head, lateral; **C**, female head, lateral. Scale line = 0.2 mm.

**Figure 2. F2:**
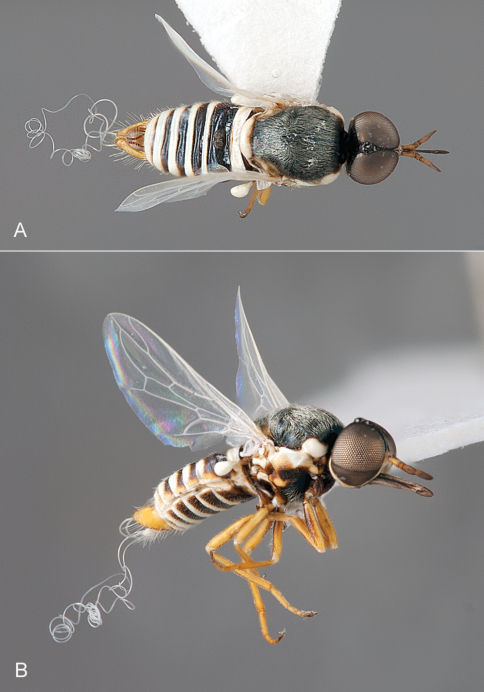
*Iranotrichia insolita* sp. n.: A. male, dorsal view [Morphbank 693172]; **B**, same, anterolateral view [Morphbank 693173]. Body length = 4.0 mm.

**Figure 3. F3:**
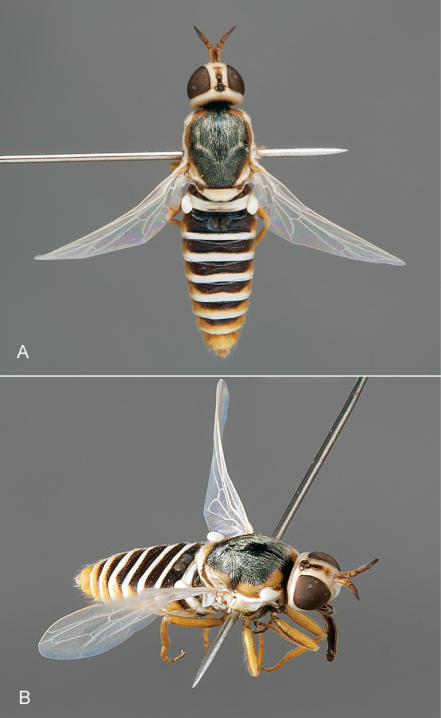
*Iranotrichia insolita* sp. n.: A. female, dorsal view [Morphbank 693174]; **B**, same, anterolateral view [Morphbank 693175]. Body length = 4.6 mm.

*Body length:* 4.0–5.5 mm [male], 4.5–5.0 mm [female]. Head higher than long, sub-spherical, female with broad, raised postocular ridge; antenna elongate, cylindrical, length 0.6–1.2× head length; antennal style subterminal, flagellum broadly rounded to truncate apically, not notched; frons relatively flat, not protruding anteriorly; mouthparts greater than head length, projecting anteriorly; scutum with dense pile of semi-appressed, silver-white lanceolate setae, all directed towards a single posteromedial point (Figs 2–3, 6); wing vein M_1_ joining with R_5 _(Fig. 1A), cell r_5_ petiolate to wing margin; wing vein M_2_ absent; costal margin ending at vein R_5_; abdomen broad, width equal to thorax; tergite 2 sensory setae well defined and two circular patches. Male genitalia: rotated 180°; tergite 7 and sternite 7 broad and separate, not ring-like; male epandrium split medially as two sclerites, halves sub-circular or sub-triangular;epandrium not covering gonocoxites ventrally; hypandrium as, paired sclerites, narrow paddle-shape with short setae along posterior margin; gonostylus well developed, irregular shaped, dark sclerotized and irregular spinose marginally; aedeagus protruding anteriorly from epandrium only a relatively short distance; gonocoxite irregularly shaped and mostly reduced; gonocoxal apodeme relatively thickened, broadly triangular with medial braces joining with aedeagus; aedeagus with lateral aedeagal bulb present, sometimes well developed; distiphallus bifid, recurved dorsally at base then greatly elongate and coiled. Female genitalia: tergite 9+10 narrow and band-like, acanthophorite spines absent; sternite 8 slightly longer than tergite 8, broadly acuminate posteriorly; furca a dark-sclerotized ring with narrow posterolateral arm, connected posteromedially with ‘Y’-shaped sclerotized bridge between furca and anterior margins of tergite 9+10; two sclerotized spherical spermathecae; spermathecal sac simple, connected to bursa medially immediately anterior to spermathecal ducts.

#### Etymology.

The genus name is derived from the type location of members of this genus; Iran, -*trichia* (Greek: hair), referring to the setal pattern and has frequently been used historically to formulate to scenopinid generic names.

**Included species.** *Iranotrichia insolita* sp. n., *Iranotrichia nigra* sp. n.

#### Comments.

This genus is placed in Scenopininae based on the rotated genitalia, wing venation and shape of the tergite two setal patches. The general habitus of members of this new genus is very reminiscent of certain genera of Bombyliidae. *Iranotrichia* gen. n. is morphologically very similar to *Metatrichia* and *Pseudomphrale*, but can be separated from these and other Scenopinidae based on the greatly elongate and often coiled bifid distiphallus, elongate antennae and mouthparts, flagellum not notched apically and with style subapical on outer surface. The elongate antennae and mouthparts and distiphallus are characteristic of *Iranotrichia*; although such elongation of these structures is sometimes found in proratine genera such as *Prorates* and *Jackhallia*, it is not found elsewhere in Scenopininae. Some species of *Pseudomphrale* and *Metatrichia* have elongated mouthparts (e.g. *Metatrichia palaestinensis* (Kröber, 1937), *Metatrichia freidbergi* Krivoshiena and Krivosheina, 1999, and *Pseudomphrale longirostris* Becker, 1913) (Krivosheina and Krivosheina 1999; Kelsey 1969) about half as long as species of *Iranotrichia* gen. n. Possibly of little phylogenetic significance, but useful for differentiating *Pseudomphrale* from *Iranotrichia* gen. n. and *Metatrichia* is that all specimens of *Pseudomphrale* species are between 1.6 and 4.0 mm body length, while specimens of *Iranotrichia* and *Metatrichia* are rarely less than 4.0 mm (Kelsey 1969; Krivosheina and Krivosheina 1996, 1999; Winterton and Woodley 2009). A greatly elongate, coiled distiphallus is also present in *Metatrichia palaestinensis* (*cf*. Kröber 1937 and Kelsey 1969). Both species of *Iranotrichia* gen. n. are known only from a single collecting event in Ghazvin province, Iran where *Iranotrichia insolita* sp. n. is apparently far more abundant than *Iranotrichia nigra* sp. n.

#### Key to Iranotrichia gen. n.

**Table:** 

1	Head, thorax, abdomen and legs with extensive areas of yellow and white; terminalia yellow; distiphallus of male as long or longer than body when uncoiled	***Iranotrichia insolita*sp. n.**
–	Head, thorax, abdomen and legs dark, with limited areas of yellow and white; terminalia dark; distiphallus of male less than length of body when uncoiled	***Iranotrichia nigra*sp. n.**

### Iranotrichia insolita sp. n.

urn:lsid:zoobank.org:act:1567D17A-C21B-48F9-9029-F451F6A9A603

http://species-id.net/wiki/Iranotrichia_insolita

Figs 1 [Fig F2] [Fig F3] 4A, B 5 

#### Type material.

**Holotype** male, IRAN: **Ghazvin province:** 17 km NE Ghazvin, Abazar village road, rangeland, 36.2916°, 50.1583°, white pan trap, 19.vi.2010, B. Gharali (NMNH). (excellent condition).

#### Paratypes.

IRAN: 55 males, 9 females, **Ghazvin province:** 17 km NE Ghazvin, Abazar village road, rangeland, 36.2916°, 50.1583°, white pan trap, 19.vi.2010, B. Gharali (CSCA (3 males), CAS (10 males 2 females), NMNH (10 males, 2 females), IRIPP (10 males, 2 females), personal collection of BG (20 males, 3 females)).

#### Diagnosis.

Head, thorax, abdomen and legs with extensive areas of yellow and/or white; combined length of scape and pedicel equal to length of flagellum; scutellum white with yellow suffusion anteromedially; terminalia yellow; epandrium elongate and sub-triangular; distiphallus of male as long or longer than body when uncoiled; distiphallus arms without spinose process.

#### Description.

**Figure 4.**
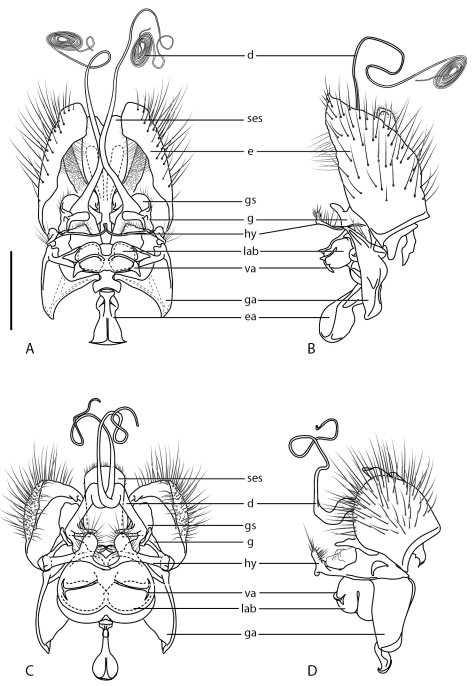
*Iranotrichia* spp. Male genitalia: **A** *Iranotrichia insolita* sp. n.: dorsal view **B** same, lateral view **C** *Iranotrichia nigra* sp. n.: dorsal view **D** same, lateral view. Scale line = 0.2 mm. Abbreviations: *d*, distiphallus; *e*, epandrium; *ea*, ejaculatory apodeme; *g*, gonocoxite; *ga*, gonocoxal apodeme; *gs* gonostylus; *ses*, subepandrial sclerite; *hy*, hypandrium; *lab*, lateral aedeagal bulb; *va*, ventral apodeme of parameral sheath.

**Figure 5.**
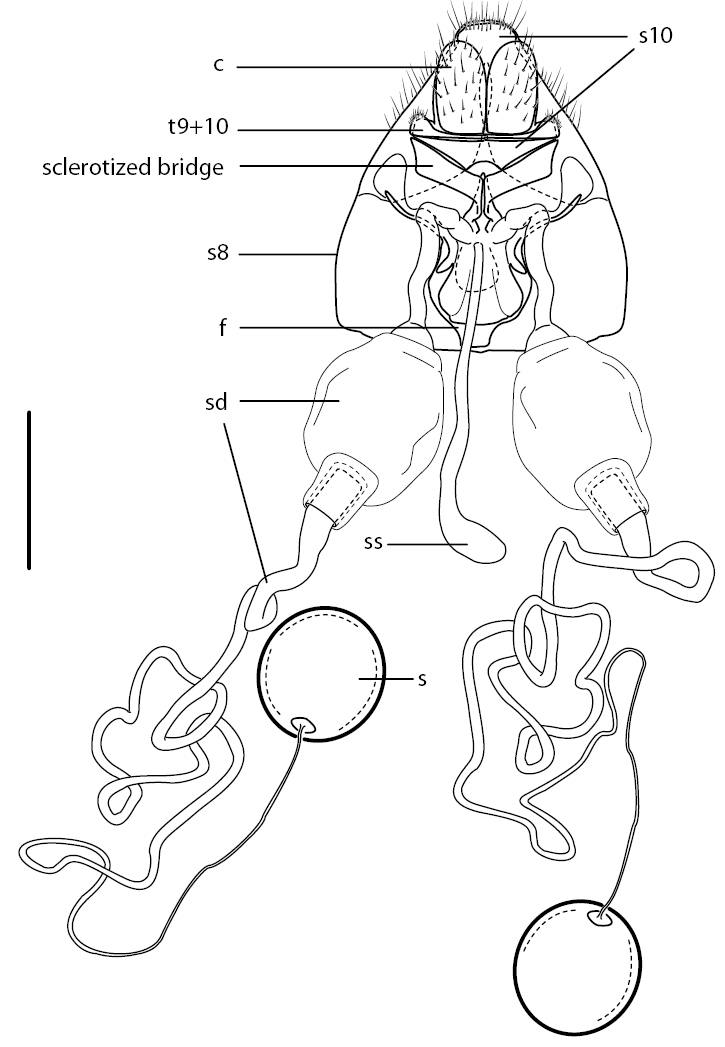
*Iranotrichia insolita* sp. n.: Female genitalia:lateral view, with tergite 8 cut away. Scale line = 0.2 mm. Abbreviations: *c*, cercus; *f*, furca; *s*, spermatheca; *sd*, spermathecal duct; *ss*, spermathecal sac; *s8*, sternite 8; s*10*, sternite 10; *t8*, tergite 8; *t9+10*, tergites 9 and 10.

Body length:4.0–4.5 mm [male], 4.5–5.0 mm [female]. *Head*. Male frons glossy black, dark yellow around base of antennae; female frons white with broad dark brown stripe medially extending ventrally from ocellar tubercle, suffused near base of antennae; frons of both sexes with short setae, setae white near base of antennae; ocellar tubercle black, raised in male, flat in female; occiput glossy black (male) or white-yellow with black medially around occipital foramen (female); occiput with sparse, short yellowish setae; gena yellow, raised as ridge along eye margin, sparse short pale setae; parafacial white to yellow, oral cavity with dark yellow sclerotized plates either side of dark medial strip; mouthparts elongate, dark brown, labellum narrow, proboscis flattened laterally (in dried specimen); palpus dark brown, short; antenna slightly longer than head length, dark yellow basally, dark brown distally; short white setae on scape and pedicel; scape 2–3X pedicel length, combined scape and pedicel length equal to length of flagellum; flagellum truncated apically. *Thorax*. Scutum black, pale white to dark yellow areas marginally (i.e. postpronotal lobe and post-alar callus), more extensive in female and additionally with yellow on anterior part of scutum adjacent to postpronotum and medially on posterior part of scutum; scutal pile dense with three indistinct vittae anteriorly on prescutum formed by unidirectional parting of setae; scutellum matte white, yellow anteromedially, sparse pale setae covering marginally; pleuron black with white to dark yellow dorsally on anepisternum and katepisternum, and around base of wing (pale area more extensive in female); white setae on katepisternum; coxae black to brown; legs yellow, femora frequently with brown suffusion basally on posterior surface; short pile of white-yellow setae on legs, longer on posterior surface of femora; distal tarsomeres suffused with brown; haltere stem brownish, knob white; wing milky hyaline from sparse microtrichia; venation cream-white. *Abdomen*. Glossy black, each segment with dark yellow laterally and thick white band along posterior margin, segments 6-8 with dark yellow more extensive along posterior margin, replacing white band; white setae on all segments, longer laterally; terminalia dark yellow with long pale setae. *Male genitalia* (Fig. 4A–B). Epandrium lobes elongate and sub-triangular, dark sclerotized margins around bases of cerci; subepandrial sclerite extending posteriorly beyond cerci, emarginate posteriorly; hypandrium lobes relatively small and paddle-like with posterior margin of setae; gonocoxite with dark sclerotized, dorsal process immediately ventral to subepandrial sclerite; gonocoxal apodeme broadly flattened, curved medially; ejaculatory apodeme relatively elongate, directed anteriorly; lateral aedeagal bulbs round; distiphallus extremely elongate, arms thick and separate basally, recurved dorsally, arms proximal before end of epandrium, distal portion greatly narrowed and highly coiled, easily longer than body length when uncoiled. *Female genitalia* (Fig. 5A). Sternite 8 with posterior edge broadly acuminate; spermathecal ducts with valves associated with large membranous sacs.

#### Etymology.

The specific epithet is derived from the Latin, *insolitus*– unusual, strange, and refers to the unusual appearance of this species.

#### Comments.

*Iranotrichia insolita* sp. n. is a highly distinctive species with contrasting black and white-yellow markings; characteristics, which differentiate this species from *Iranotrichia nigra* sp. n., among others, include the extremely elongate distiphallus and lack of spinose processes at the base of the distiphallus. The antennae and mouthparts are the longest of any scenopinid and are presumably associated with feeding at flowers.

### Iranotrichia nigra sp. n.

urn:lsid:zoobank.org:pub:066C7733-FC95-4532-9465-8B123D0BEB33

http://species-id.net/wiki/Iranotrichia_nigra

Figs 4C–D 6 

#### Type material.

#### Holotype

 male, IRAN: **Ghazvin province:** 17 km NE Ghazvin, Abazar village road, rangeland, 36.2916°, 50.1583°, white pan trap, 19.vi.2010, B. Gharali (NMNH). (excellent condition).

#### Paratype.

IRAN: **Ghazvin province:** 1 male, 17 km NE Ghazvin, Abazar village road, rangeland, 36.2916°, 50.1583°, white pan trap, 19.vi.2010, B. Gharali (personal collection of BG/IRIPP)

#### Diagnosis.

Head, thorax, abdomen and legs black, with limited areas of yellow; combined length of scape and pedicel approximately 2/3 length of flagellum; scutellum black with yellow-white marginally; male terminalia dark; epandrium sub-circular; distiphallus of male sub equal to length of abdomen when uncoiled; distiphallus arms with spinose process basally.

#### Description.

Body length:4.5 mm [male]. *Head*. Male frons glossy black, dark yellow below base of antennae, short white setae above base of antennae; ocellar tubercle black, raised in profile; occiput glossy black with sparse, short yellowish setae; gena cream-white, raised as ridge along eye margin, sparse short pale setae; parafacial white to yellow with brown suffusion; oral cavity with dark yellow sclerotized plates either side of dark medial stripe; mouthparts elongate, dark brown, labellum narrow, proboscis flattened laterally (in dried specimen); palpus short, dark brown; antenna 0.6× head length, uniform dark brown; short white setae on scape and pedicel; scape 2× pedicel length, combined scape and pedicel length less than length of flagellum; flagellum tapered slightly apically. *Thorax*. Scutum black, yellow areas marginally (postpronotal lobe and post-alar callus); scutal pile dense; scutellum black, yellow-white marginally; pleuron black with white suffusion dorsally on anepisternum, and around base of wing; white setae on anepisternum and katepisternum; coxae black to brown with white setae; legs black, yellow apically on femora, basally on tibiae and basitarsi; short pile of white-yellow setae on legs, longer on posterior surface of femora; haltere stem brownish, knob white; wing milky hyaline from sparse microtrichia; venation cream-white. *Abdomen*. Glossy black, each segment with a thick white band along posterior margin, segments 6-8 with dark yellow more extensive along posterior margin, replacing white band; white setae on all segments, longer laterally; terminalia black with dark brown on epandrium, with long pale setae. *Male genitalia* (Fig. 4C–D). Epandrium lobes rounded and sub-circular, dark sclerotized margins around bases of cerci; subepandrial sclerite quadrangular; hypandrium lobes relatively well developed, paddle-like narrower anterior medial process, posterior margin with pale setae; gonocoxite dark sclerotized, with dorsal process immediately medial to subepandrial sclerite; gonocoxal apodeme very broadly, curved medially; ejaculatory apodeme spatulate, directed anteriorly; lateral aedeagal bulbs relatively large, each subdivided dorsoventrally into two chambers; distiphallus elongate, arms thick and separate basally, recurved dorsally, with ventromedially directed spinose process at base, arms overlap before end of epandrium, distal portion narrowed and coiled, not longer than body length when uncoiled.

**Figure 6.**
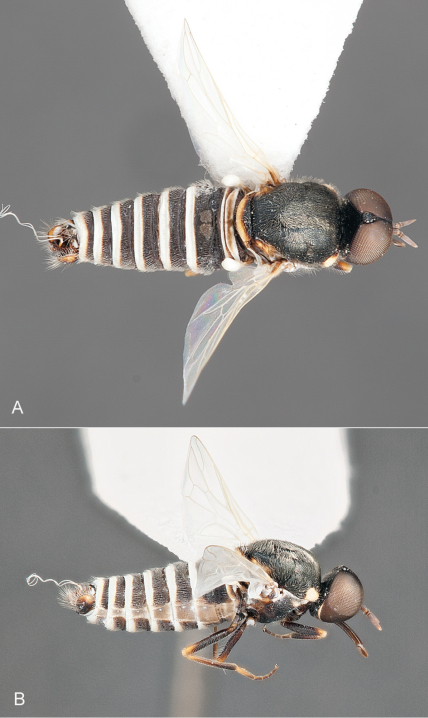
*Iranotrichia nigra* sp. n.: **A** male, dorsal view [Morphbank 693176] **B** same, lateral view [Morphbank 693177]. Body length = 4.5 mm.

#### Etymology.

The specific epithet is derived from the Latin, *nigra*– black, dark, and refers to the overall dark colour of this species.

#### Comments.

*Iranotrichia nigra* sp. n. is differentiated from *Iranotrichia insolita* sp. n. by the shorter antennae, rounded epandrial lobes, shorter male distiphallus, secondarily subdivided lateral aedeagal lobes and presence of spinose processes at the base of the distiphallus. The female of this species is unknown.

### Kelseyana nom. n.

Caenoneura  Kröber, 1924: 75. – Thomson 1870: 270. – Kirby 1890: 136. – Kelsey 1969: 162. – Hassan and El-Hawagry 2001: 2.

#### Type species

*Caenoneura robusta* Kröber, 1924: 75.

#### Included species.

*Kelseyana nigra* (Kelsey, 1969) comb. n., *Kelseyana robusta* (Kröber, 1924) comb. n.

## Supplementary Material

XML Treatment for Iranotrichia

XML Treatment for Iranotrichia insolita

XML Treatment for Iranotrichia nigra

XML Treatment for Kelseyana
